# Our endeavor to write a new chapter in the development of laboratory animal sciences

**DOI:** 10.1002/ame2.12211

**Published:** 2022-02-15

**Authors:** Jian‐Dong Jiang

**Affiliations:** ^1^ Academician of Chinese Academy of Engineering Editor‐in‐Chief of Acta Pharmaceutica Sinica B Chairman, Department of Pharmacology Peking Union Medical College & Chinese Academy of Medical Sciences Beijing China

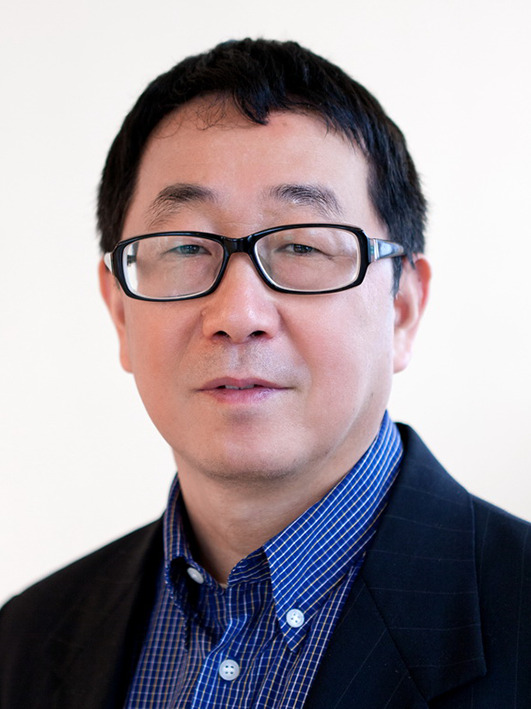

Since the beginning of the 21st century, with the wide application of molecular biology and genetic engineering technology, the more holistic discipline of laboratory animal sciences has also developed rapidly and many excellent innovations based on laboratory animals, animal models and experimental medicine have continued to emerge. *Animal Models and Experimental Medicine (AMEM)* was launched in 2018, which was timely and attracted the attention of the industry. Four years have passed and *AMEM* has achieved fruitful results. Here, I would like to extend my hearty congratulations to the journal.

From ancient times to the present, laboratory animal sciences have played a pivotal role in our understanding of life and prevention and treatment of diseases. Over recent centuries, through animal experiments, humans learned about blood circulation, leading to the independent discipline of physiology, and vaccines were invented, ridding us of the nightmare of deadly infectious diseases. Focusing on the present, we find that laboratory animal sciences have become an important indicator of the developmental level of biomedicine in a country or region. In a very recent study, xenogeneic organs (animal organs) were successfully transplanted to patients, setting a new frontier in the treatment of human diseases. Looking to the future, we believe that the multidisciplinary intersection, integration and innovative development of laboratory animal sciences will continue to make key contributions to our understanding of the mysteries of human life, the mechanism of diseases and the maintenance of human health.

*AMEM* is an international open access, peer‐reviewed journal dedicated to disseminating research and contemporary information in the field of laboratory animal sciences. Topics explored by *AMEM* include new animal models of human diseases, laboratory animals, laboratory animal welfare, laboratory animal biotechnology, as well as applied research in experimental and translational medicine.

Over the past four years, *AMEM* has published original articles on animal models in many fields, demonstrating the journal's professional focus on laboratory animal models. These new animal models provide important support for research in life sciences, medicine, pharmacy, food, environment and aerospace. For example, the recent launch of COVID‐19 rhesus monkey model has promoted research into COVID‐19 vaccines and therapeutic drugs.^1^

At the same time, *AMEM* focuses on experimental medicine, comparative medicine and translational medicine, highlighting the advantages of research at the intersection and integration of laboratory animal disciplines. *AMEM* also attaches great importance to laboratory animal welfare and has added a special section on Laboratory Animal Welfare and Ethics.

As we begin a new year, I believe that *AMEM* will continue to make further progress. Innovation‐driven development and interdisciplinary intersection and integration are the characteristics of scientific research in this era. The deep integration of laboratory animal sciences with medicine, pharmacy, biology, etc. will surely build a bridge between basic research and clinical research, promote the effective transformation of animal and human data into therapies, and accelerate the process of new drug research and development. My sincere wish is that *AMEM* will continue to champion the benefits of multidisciplinary intersection and integration in the field of laboratory animal sciences, promote the deep integration and innovative development of related disciplines in a multi‐faceted manner, accelerate the transformation of data to clinical achievements and create a world‐class journal with distinctive characteristics.
